# Environmental characteristics drive variation in Amazonian understorey bird assemblages

**DOI:** 10.1371/journal.pone.0171540

**Published:** 2017-02-22

**Authors:** Juliana Menger, William E. Magnusson, Marti J. Anderson, Martin Schlegel, Guy Pe’er, Klaus Henle

**Affiliations:** 1 UFZ – Helmholtz Centre for Environmental Research, Department of Conservation Biology, Leipzig, Saxony, Germany; 2 Faculty of Biosciences, Pharmacy and Psychology, University of Leipzig, Leipzig, Saxony, Germany; 3 Coordenação de Pesquisa em Biodiversidade, Instituto Nacional de Pesquisas da Amazônia - INPA, Manaus, Amazonas, Brazil; 4 New Zealand Institute for Advanced Study - NZIAS, Albany Campus, Massey University, Auckland, New Zealand; 5 German Centre for Integrative Biodiversity Research (iDiv) Halle-Jena-Leipzig, Leipzig, Saxony, Germany; Universidade Federal de Goias, BRAZIL

## Abstract

Tropical bird assemblages display patterns of high alpha and beta diversity and, as tropical birds exhibit strong habitat specificity, their spatial distributions are generally assumed to be driven primarily by environmental heterogeneity and interspecific interactions. However, spatial distributions of some Amazonian forest birds are also often restricted by large rivers and other large-scale topographic features, suggesting that dispersal limitation may also play a role in driving species’ turnover. In this study, we evaluated the effects of environmental characteristics, topographic and spatial variables on variation in local assemblage structure and diversity of birds in an old-growth forest in central Amazonia. Birds were mist-netted in 72 plots distributed systematically across a 10,000 ha reserve in each of three years. Alpha diversity remained stable through time, but species composition changed. Spatial variation in bird-assemblage structure was significantly related to environmental and topographic variables but not strongly related to spatial variables. At a broad scale, we found bird assemblages to be significantly distinct between two watersheds that are divided by a central ridgeline. We did not detect an effect of the ridgeline *per se* in driving these patterns, indicating that most birds are able to fly across it, and that differences in assemblage structure between watersheds may be due to unmeasured environmental variables or unique combinations of measured variables. Our study indicates that complex geography and landscape features can act together with environmental variables to drive changes in the diversity and composition of tropical bird assemblages at local scales, but highlights that we still know very little about what makes different parts of tropical forest suitable for different species.

## Introduction

Understanding processes that drive spatio-temporal changes in species richness, abundance and composition is a central objective of community ecology. Several theories have been advanced to explain how so many species can coexist in megadiverse tropical forests. While niche theory suggests that species’ distributions are driven by environmental heterogeneity and species’ interactions [1–4], neutral theory posits that species’ distributions arise largely from random processes, with local assemblage composition being determined mainly by stochastic processes and dispersal limitation [5]. Empirical studies attempting to disentangle the relevance of niche vs. neutral processes in shaping the composition of tropical assemblages have shown complementary effects of environmental factors and dispersal limitation [6–12], with their relative importance often depending on the spatial scale of the study.

Many previous studies have used geographical distance as a proxy for dispersal limitation, without explicitly modeling potential effects of physical landscape features that may act as barriers to species’ movement [9]. For instance, in lowland Amazonia, large rivers have been identified as barriers to dispersal of several vertebrate species [13]. At large scales, some Amazonian forest birds have distributions restricted to distinct areas of endemism, delimited primarily by the major Amazonian rivers [14–19]. At finer scales, there is growing evidence that Amazonian forest birds have restricted dispersal [20–23]. Local landscape features, such as mosaics of inhospitable areas and even narrow roads through a forest, may act as barriers, limiting bird territories and movements [24–26].

Nonetheless, within an interfluve, variation in Amazonian forest bird communities is usually assumed to be driven primarily by environmental heterogeneity and interspecific interactions, rather than by dispersal limitation [4, 27]. Indeed, most Amazonian forest birds are thought to be habitat specialists, and their spatial patterns of diversity and composition are affected by environmental factors, such as vegetation structure, floristic composition and topography [28–34]. Although the role of dispersal traits in structuring avian assemblages in fragmented landscapes has been investigated [35], few studies have simultaneously investigated the effects of environmental characteristics and dispersal limitation on Amazonian bird-assemblages in naturally heterogeneous landscapes (but see [9, 31]).

**Fig 1:**
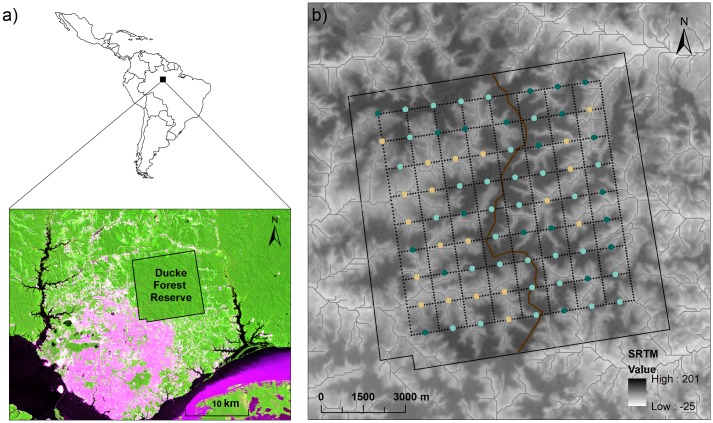
Location of Ducke Forest Reserve (DFR), Manaus, Amazonas State, Brazil. Location of DFR in relation to the city of Manaus and to the main rivers (a); Topography, streams and the system of trails (dashed lines) in the study area, showing the 72 sampling plots (b). The brown line marks the division of the reserve into eastern (*n* = 34) and western (*n* = 38) watersheds. The colors of the plots indicate the three environmental groups identified by K-means partitioning. Beige dots represent Low areas (*n* = 20), pale green dots represent High areas (*n* = 31), and dark green dots represent Slope areas (*n* = 21).

Here, we investigate the effects of environmental characteristics, landscape features and spatial variables on variation in the diversity and structure of bird assemblages in a 10,000 ha reserve in central Amazonia, Brazil. We sampled understorey birds in the Ducke Forest Reserve (DFR, Fig 1), which is covered by largely undisturbed old-growth forest. Although the urban sprawl of the city of Manaus has reached the southern and western limits of DFR, the reserve is still connected to continuous forest on its eastern side and does not show any obvious impacts of urbanization within its limits. Small streams are abundant in the area, resulting in an undulating terrain with ridges up to 140 m above sea level (a.s.l.), interspersed by valleys that may be as low as 40 m a.s.l., yielding high environmental variation [36]. Moreover, the reserve is traversed by a central ridge which runs from north to south, dividing DFR into two major watersheds: the western watershed drains to tributaries of the Negro River (black water), while the eastern watershed drains to tributaries of the Amazon River (white water). The spatial configuration of DFR provides an excellent test-bed for the evaluation of environmental, topographic and spatial factors affecting forest-bird assemblage structure at a local scale.

Despite its small elevational range, we hypothesized that the central ridgeline reduces bird movements across watersheds, contributing to distinct bird assemblages in each watershed. We investigated spatiotemporal variation in diversity and assemblage structure and quantified the extent to which environmental, topographic and spatial variables explain these patterns.

## Methods

### Ethics statement

Fieldwork was carried out with authorization and approval of the Brazilian Biodiversity Authorization and Information System—SISBIO (Permit 34850) and of the Brazilian Center for Bird Banding and Conservation—CEMAVE (Permit 3576). These licenses covered all necessary animal ethics, including a permit to capture birds, and appropriate methods for handling and banding the birds, in accordance with the environmental legislation of Brazil (Instrução Normativa IBAMA N° 27/2002 and Instrução Normativa ICMBio N° 154/2007); following protocols established by CEMAVE [37]. We affirm fieldwork did not involve endangered or protected species.

### Study site

The Ducke Forest Reserve (02°55’– 03°01’S, 59°53’– 59°59’W), located on the outskirts of Manaus city, Amazonas state, Brazil (Fig 1a), is covered by *terra firme* forests that are not subject to long-term floods [36]. The understorey is dominated by acaulescent palms and shaded by a closed canopy. Soil type varies along an elevation gradient; soils with higher clay content occur at higher elevation and sandier soils occur at lower elevation [38]. The mean annual temperature and precipitation from 1979 to 2008 were 26°C and 2524 mm, respectively [39]. A rainy season typically occurs from November to June and a dry season from July to October [36].

DFR is a site in the Brazilian Long Term Ecological Research (LTER) network and has a systematic sampling grid that is part of the Brazilian Biodiversity Research Program (PPBio, https://ppbio.inpa.gov.br). The grid gives access to 72 permanent plots placed systematically at 1-km intervals along nine east-west trails (Fig 1b). Each plot is 250 m in length and follows topographic contour lines to avoid within-plot edaphic variation [40, 41].

### Bird sampling

We sampled birds in all 72 plots of the DFR during the dry season in each of three consecutive years (2012–2014). To avoid biases in capture rates due to net avoidance [42], each plot was sampled on a single day during each sampling period. Sixteen mist-nets (each 9 m long, 32 mm mesh size) were set in pairs at 10-m intervals along the 250 m length of each plot [43]. Mist-nets were opened between 06:00 and 12:00 and inspected every 40 min. Birds captured were identified and banded with metal bands issued by CEMAVE. The total abundance of each species captured in each netting event was aggregated per plot. Mist-netting is a widely used technique to sample understorey birds, as it detects more cryptic, ground-foraging and non-singing birds than aural or visual surveys [28]. However, it is also known to under-sample species which usually fly above net level, and only occasionally descend to the ground [32]. To evaluate whether under-sampling might influence the results, we analyzed the data in two separate sets: 1) “all species” (98 spp, see Results)–which included all species captured and; 2) “common forest understorey species” (63 spp)–which included only species captured in more than two plots, that use predominantly the lower layers of the forest, and use primarily *terra firme* forests (following [44]). Bird data analyzed in this study are deposited in the PPBio, MetaCat Repository: https://ppbiodata.inpa.gov.br/#view/PPBioAmOc.82.4 [45].

### Identification of environmental groups

Data on elevation, slope, clay and silt content of the soil, tree and palm density, distance to the nearest stream and geographical coordinates (latitude and longitude) for each plot were used as explanatory variables (S1 Table, as shared on the PPBio website). Elevation, slope, distance to the nearest stream, clay and silt content of the soil, tree and palm density were normalized to z-scores, and K-means partitioning [46, 47] was used to identify groups of plots based on these environmental variables. We determined the number of groups by considering the pattern of decrease in the within-group sum of squared distances to centroids with increasing numbers of groups (K). The within-group sum of squares was calculated from 2 to 8 groups. We looked for an “elbow” in the plot to make a decision about an appropriate number of groups and considered 3, 4, 5 or 6 groups to be potentially reasonable. Principal component analysis (PCA) was used to visualize and describe the groups in terms of the environmental variables that gave rise to them. The PCA showed a clear separation of the 72 plots into three groups (S1 Fig), but other potential groupings either showed mixed symbols across the PCA space or generated individual outliers. We therefore subsequently used three groups to represent the environmental variation within DFR (Fig 1, S1 Fig). Accordingly, plots in group 1 (*n* = 20) were characterized as occurring at low elevation, being close to streams and having high palm densities (hereafter referred to as “Low areas”); plots in group 2 were characterized as occurring at high elevation (*n* = 31) and having soils with high clay and silt content (“High areas”); plots in group 3 (*n* = 21) were characterized as occurring on relatively steep slopes and having high tree densities (“Slope areas”).

### Analyses of bird richness and abundance

ANOVA was used to partition variation in each of two variables: number of bird species captured per plot and log total abundance according to three factors: ‘Year’ (fixed with three levels: 2012, 2013 and 2014), ‘Watershed’ (fixed with two levels: eastern and western) and ‘Environmental group’ (fixed with three levels: Low areas, High areas, and Slope areas). We tested for interaction effects and used Tukey’s HSD tests for *a posteriori* pairwise comparisons. These analyses were carried out using the R statistical program [48].

### Analyses of bird assemblage structure

#### Comparisons among years, watersheds and environmental groups

Permutational multivariate dissimilarity-based ANOVA (PERMANOVA [49, 50]) was used to partition variation in bird assemblage structure on the basis of a zero-adjusted Bray-Curtis dissimilarity matrix [51] calculated from square-root transformed abundances. A three-factor PERMANOVA (with the factors ‘Year’, ‘Watershed’, and ‘Environmental group’) was carried out. *P*-values for all main effects, interaction terms and *a posteriori* pairwise comparisons were obtained using 9999 permutations of residuals under a reduced model [52, 53]. To visualize patterns of differences among multivariate centroids, we constructed metric multi-dimensional scaling (mMDS) plots of 100 bootstrap means with 95% confidence regions [54].

A compound graph [55] was used to characterize the spatial turnover of individual bird species across the two watersheds. In addition, canonical analysis of principal coordinates (CAP [56, 57]) was used to model the three environmental groups on the basis of the dissimilarity matrix, and leave-one-out misclassification error [58] was used to determine the number of principal coordinate (PCO) axes (*m*) to use for the CAP model and also to measure the distinctiveness of each of the groups. Vectors corresponding to raw Pearson correlations of individual bird species variables with each of the resulting CAP ordination axes were used to characterize the avifauna associated with each *a priori* environmental group. All CAP and PERMANOVA analyses were done using PRIMER v7 [54] with the PERMANOVA+ add-on package [59].

#### Relationships with environmental, topographic and spatial predictor variables

To relate variation in bird assemblage structure with environmental, topographic and spatial predictor variables, abundance data were first summed within plots across years. We considered three different groups of predictor variables: (i) those variables that directly characterized environmental conditions (i.e., clay, silt, tree density, palm density and distance to the nearest stream); (ii) those variables that identified geographical features having three-dimensional structure (i.e., elevation, slope and watershed—a single variable coding the contrast (+1, -1) of eastern vs. western watersheds); and (iii) purely spatial variables, consisting of latitude (*y*) and longitude (*x*), which for simplicity were each scaled to a range of 0–10, along with their polynomials up to 3^rd^ order (e.g., [60]). Although topographic variables such as slope and elevation tend to be correlated with other (measured and unmeasured) environmental variables, they do in fact correspond, strictly speaking, to structural measures of the landscape.

The rationale for the approach we took to relate bird assemblages with potential predictor variables had three important features: (i) we allocated predictor variables into subsets that were directly aligned with *a priori* hypotheses concerning potential drivers of biotic variation in the birds; (ii) the methodology used to identify an appropriate *number* of variables that might usefully be included in parsimonious models (either within each subset or overall) was achieved using non-arbitrary sequential conditional permutation tests; and (iii) the “best” model (once again, either within subsets or overall) was identified using an information criterion approach. Note that step (ii) was *not* used to identify a “best” model, nor to identify *which* particular variables should be included in such a model.

Our analyses had two aims. First, we wished to compare the relative importance, overlap and strength of the association between each of these three sets of predictor variables (environmental, topographic and spatial) and the bird assemblages. Second, we wished to find a parsimonious model using all potential predictor variables individually and taking into account their correlations. For the first aim, we began by obtaining a parsimonious subset of variables separately for each of the three sets using forward selection and sequential conditional distance-based redundancy analysis (dbRDA [50, 61]) tests (with 9999 permutations under a reduced model [52, 53]) to explain the variation in the zero-adjusted Bray-Curtis dissimilarity matrix of square-root-transformed bird-assemblage data. A *P*-value that exceeded 0.10 was used as a cut-off in the suite of sequential conditional tests to identify the number of variables (*q*) that would be sensible to retain. We then implemented the DISTLM model-selection tool in PERMANOVA+ for PRIMER v7 [59] to find the best *q*-variable subset *within* each set, based on a direct multivariate analogue to the small-sample-corrected Akaike information criterion (AICc [59, 62]). The parsimonious environmental, topographic and spatial subsets obtained were then used to make comparisons *among* these three sets. This was done by comparing their AICc values directly and also by doing forward selection and associated sequential conditional tests of these three subsets (For further details concerning the fitting and selection of whole sets of variables using DISTLM, see [59]).

For the second aim, we first examined the Pearson correlations among all pairs of variables. We then used dbRDA to model the relationship between bird assemblage structure and all of the predictor variables, as follows. First, each variable was separately tested for its individual relationship with bird assemblage structure (ignoring other variables) in a series of marginal tests, each with 9999 permutations. Next, we applied a forward-selection with sequential conditional tests using DISTLM to identify the number of variables (*q*) that might sensibly be included in a parsimonious model, considering (as before) a cut-off of *P* > 0.10 in the sequential tests to identify *q*. Finally, the best *q*-variable model was identified on the basis of the direct multivariate analogue to AICc in order to obtain an overall parsimonious model, whose fitted values were then visualized using dbRDA [59]. The model resulting from the above procedure was also compared with the model that would have been obtained using a direct, unconstrained and exhaustive search over all possible predictor variables on the basis of the AICc criterion.

## Results

### Bird richness and abundance

We captured 2483 birds belonging to 98 species, including *Cacicus solitarius* Vieillot 1816, a new record for DFR (S2 Table). The mean number of individuals captured per plot per year was 11.5 (± 0.43 SE) and ranged from 2 to 42. The mean number of species captured per plot per year was 7.57 (± 0.23 SE) and ranged from 2 to 18. The 21 most-captured species (each having a total abundance of ≥ 30 individuals captured across the three time periods) accounted for 77% of all captures. Twenty-three species were captured only once. Recapture rate was 7%; most individuals were recaptured in the same plot they were originally captured, and 35 species were recaptured at least once over the three years of study.

**Fig 2:**
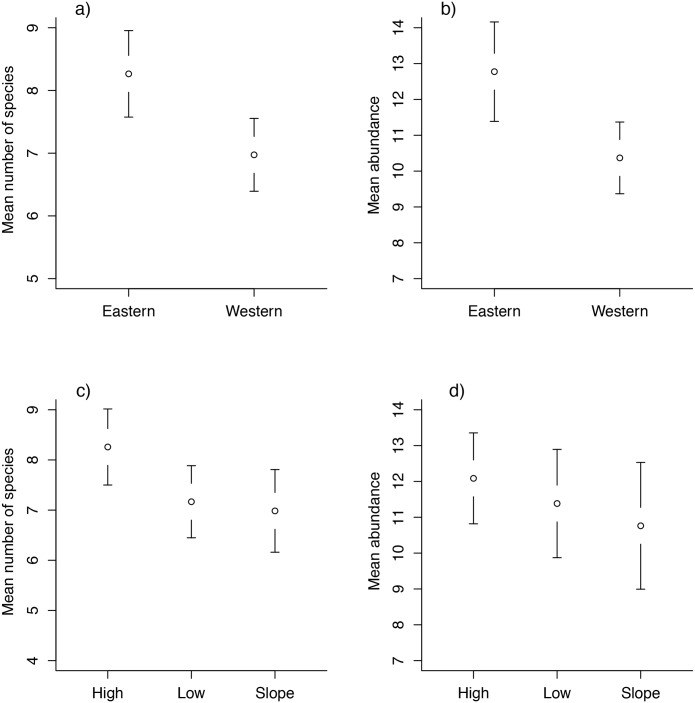
Number of species and abundance of birds vs. environmental groups and watersheds. Mean number of species captured per plot or mean abundance in different watersheds (a and b, respectively) and in different environmental groups (c and d, respectively). Bars indicate 95% confidence intervals.

Our analyses with the two separate sets of bird data (all species and common forest understorey species) yielded similar results, thus we show results using all 98 captured species (see S1 File for results obtained using the other subset). Mean number of species captured per plot and log abundance of birds did not vary significantly over the three years of sampling (*F*_2_ = 1.602, *P* = 0.204, and *F*_2_ = 1.450, *P* = 0.237, respectively), but did differ significantly between watersheds (*F*_1_ = 8.255, *P* = 0.005; *F*_1_ = 8.775, *P* = 0.003, respectively), with a greater average number of species and log abundance of birds occurring in the eastern than in the western watershed (Fig 2a and 2b, respectively). There were significant differences among the environmental groups in mean number of species captured (*F*_2_ = 3.447, *P* = 0.034), but not in mean log abundance (*F*_2_ = 1.679, *P* = 0.189) (Fig 2c and 2d, respectively). No significant interactions were detected between any of the factors (all *P* > 0.1). Tukey’s HSD tests indicated plots located in High areas had a significantly higher mean number of species captured (*P* = 0.05) than plots in Slope areas; no other pairwise comparisons were statistically significant (*P* > 0.1).

### Bird assemblage structure

#### Comparisons among years, watersheds and environmental groups

**Fig 3:**
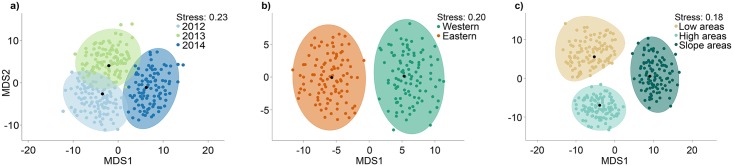
Ordinations of bootstrap averages of bird assemblages. Two-dimensional metric multi-dimensional scaling ordinations of 100 bootstrap sample averages for each group for each of the main factors: Years (a), Watersheds (b); and Environmental groups (c), showing the overall mean (black dots) and the empirical approximate 95% confidence region, based on zero-adjusted Bray-Curtis dissimilarities of square-root transformed abundances of 98 bird species.

There were significant differences in bird composition among years (PERMANOVA pseudo-*F*_*2*_ = 1.515, *P* = 0.042; Fig 3a); between watersheds (pseudo-*F*_*1*_ = 1.932, *P* = 0.018; Fig 3b), and among environmental groups (pseudo-*F*_*2*_ = 3.035, *P* < 0.001; Fig 3c). There were no significant interactions between any of the factors (*P* > 0.1). Pairwise tests indicated that only the bird assemblages from 2012 and 2014 differed significantly from one another (Fig 3a, S3 Table), whereas the assemblages of birds in each of the three environmental groups clearly differed significantly from one another (Fig 3c, S3 Table). Turnover in the identities of bird species between the two watersheds is shown in S2 Fig.

The High and Low areas had distinct avifaunal assemblages, each showing ~70% allocation success in the CAP model with *m* = 13 PCO axes (S3 Fig). Low areas were characterized by birds that occur more frequently in riparian habitats (e.g., *Phaethornis superciliosus*, *Campylopterus largipennis*, *Mionectes macconnelli*, *Schistocichla leucostigma* and *Onychorhynchus coronatus* [32, 43, 44]), while some mixed-species flocking birds tend to occur more frequently in High areas (e.g., *Thamnomanes* spp., *Myrmotherula* spp., *Xenops minutus*, *Deconychura longicauda* and *Hylophilus ochraceiceps* [63]). Slope areas were less distinct (only ~43% allocation success under the CAP model), but did show a greater prevalence of frugivorous birds, such as *Lepidothrix serena*, *Pseudopipra pipra*, *Corapipo gutturalis* and *Pteroglossus viridis* (S3 Fig).

#### Relationships with environmental, topographic and spatial predictor variables

Sequential tests of the environmental set of five variables indicated that *q* = 3 variables would be sufficient to capture the variation explained by this set in a parsimonious way (S4 Table). The best 3-variable model for the environmental set (based on AICc) contained the variables of distance from the nearest stream, clay and tree density, which together explained 9.24% of the variation in bird assemblages and had an AICc value of 537.76 (Table 1, marginal tests). Similarly, for the topographic set, *q* = 3 variables were identified as sufficient to explain variation for modeling purposes (S4 Table); that is, all three variables: elevation, slope and watershed, were deemed useful here, which together explained 8.93% and had an AICc value of 539.78 (Table 1, marginal tests). In contrast, for the spatial set of variables, only *q* = 1 variable was deemed relevant—all sequential tests after fitting the variable of *y*^2^ (i.e., squared latitude) had *P*-values > 0.10 (S4 Table). Squared latitude explained only 2.24% of the variation in bird assemblages, however. To allow direct comparison with the environmental and topographic sets, we determined the best 3-variable model also for the spatial set on the basis of AICc, which included the variables of *y*^2^, *x*^2^ and *x*^3^. This explained 5.0% of the variation in the bird assemblage data, with an AICc of 541.02 (Table 1, marginal tests).

**Table 1:** Results of DISTLM analyses among sets of predictors.

	MARGINAL TESTS				SEQUENTIAL TESTS		
set	pseudo-*F* (4, 68)	*P*	*R*^2^	AICc	pseudo-*F*	*P*	cumulative *R*^2^
**environmental**	2.307	**0.0001**	0.0924	537.76	2.307 (4, 68)	**0.0001**	0.0924
**topographic**	2.222	**0.0001**	0.0893	539.78	1.599 (7, 65)	**0.0027**	0.1547
**spatial**	1.199	0.1550	0.0502	541.02	1.149 (10, 62)	0.2176	0.1993

Proportion of variation (*R*^2^) in bird assemblage structure that is explained by each set of variables when taken alone (marginal tests), and the cumulative proportion explained by fitting variables sequentially using forward selection.

Values in bold indicate significant effects.

Clearly, although none of these subsets of variables explained much of the variation in bird assemblages (each 3-variable set having a *R*^2^ < 0.10), the environmental variables explained the most, followed by the topographic variables, and with the least being associated with the purely spatial variables (Table 1, marginal tests). Furthermore, the sequential conditional tests of these whole sets (Table 1, sequential tests) demonstrated that the topographic variables significantly contributed to explain variation in bird assemblages, over and above that explained by the environmental variables, to yield a cumulative *R*^2^ of 0.1547 (*P* = 0.027), whereas the addition of purely spatial variables did not (*P* = 0.2176).

When each predictor variable was considered individually, significant relationships with variation in bird assemblages were found for elevation, slope, clay, palm density, distance to nearest stream, and latitude squared (*P* < 0.05), with tree density, watershed and latitude showing marginal effects (*P* < 0.10; Table 2, marginal tests). However, a few strong correlations among variables were apparent, as was already evidenced by the PCA (see S1 Fig). Specifically, elevation and clay content had a Pearson correlation of *r* = 0.94; hence these two variables should be viewed as being instrumentally equivalent (i.e., each may act as a proxy for the other) in any model selection procedure. The next-highest correlation was between clay and distance from the nearest stream (*r* = 0.75), followed by that between elevation and distance from the nearest stream (*r* = 0.71); all other correlations were less than 0.45.

**Table 2:** Results of DISTLM analyses on all predictors.

	MARGINAL TESTS			SEQUENTIAL TESTS		
variable	pseudo-*F*	*P*	*R*^2^	pseudo-*F*	*P*	cumulative *R*^2^
**dist. stream**	2.954	**0.0001**	0.0405	2.954	**0.0001**	0.040
**elevation**	2.728	**0.0003**	0.0375	2.410	**0.0009**	0.073
**slope**	2.582	**0.0004**	0.0356	2.175	**0.0031**	0.102
**tree**	1.565	**0.0597**	0.0219	1.548	**0.0612**	0.122
***x***	1.170	0.2740	0.0164	1.484	**0.0847**	0.141
**watershed**	1.439	**0.0978**	0.0201	1.352	0.1453	0.159
**silt**	1.241	0.2130	0.0174	1.428	0.1136	0.177
**clay**	2.643	**0.0002**	0.0364	1.171	0.2760	0.192
***y***^**3**^	1.600	**0.0513**	0.0223	1.045	0.4090	0.205
***x***^**3**^	1.021	0.4361	0.0144	0.953	0.5128	0.218
**palm**	1.666	**0.0360**	0.0232	0.943	0.5276	0.230
***x***^**2**^	1.093	0.3556	0.0154	0.869	0.6221	0.241
***y***	1.588	**0.0539**	0.0222	0.773	0.7318	0.251
***y***^**2**^	1.607	**0.0462**	0.0224	0.817	0.6896	0.262
***yx***^***2***^	1.144	0.3058	0.0161	0.592	0.8972	0.269
***yx***	1.346	0.1452	0.0189	0.728	0.7853	0.279
***y***^**2**^***x***	1.251	0.2008	0.0176	1.162	0.2742	0.294

Proportion of variation (*R*^2^) in bird assemblage structure (based on adjusted Bray-Curtis dissimilarities of square-root transformed abundances) explained by each predictor variable when taken alone (marginal tests) and the cumulative proportion explained by fitting variables sequentially using forward selection. *x* and *y* refer to longitude, latitude and their polynomials up to 3rd order, respectively.

Values in bold indicate *P* < 0.1.

**Fig 4:**
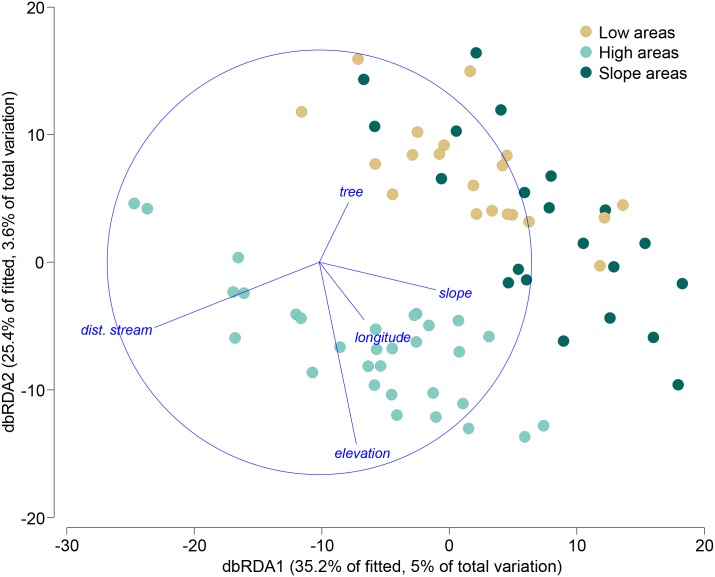
Constrained ordination relating bird data to predictor variables. Distance-based redundancy analysis (dbRDA) of zero-adjusted Bray-Curtis dissimilarities calculated from square-root transformed abundances of 98 bird species vs. elevation, slope, tree density, distance to nearest stream, and longitude (*x*), identified as the best 5-variable model using the AICc selection criterion. Colors identify the three environmental groups.

Forward selection and sequential conditional tests across all potential predictor variables indicated that a parsimonious model to explain variation in the bird assemblage data on the basis of all potential predictor variables would be achieved using *q* = 5 variables (Table 2, sequential tests). The best 5-variable AICc model included elevation, slope, tree density, distance to nearest stream, and longitude (*x*). These 5 variables explained 14.1% of the variation in the bird assemblages and the corresponding model had an AICc value of 538.47. The model was visualized with a dbRDA ordination of the fitted values (Fig 4), whose first 2 axes captured 60.59% of the fitted variation, but only 8.55% of the total variation. A direct, unconstrained and exhaustive search over all potential predictor variables on the basis of AICc yielded a model with only 3 variables: elevation, slope and distance to nearest stream, which explained 10.16% of the variation and had an AICc value of 537.02, effectively equivalent (having ΔAICc < 1.5) to the 5-variable model shown in Fig 4. Although the three environmental groupings categorized from the PCA were also clearly identifiable on the dbRDA plot, the vast majority of the variation in bird assemblages (> 85%) remained unexplained (Table 2, sequential tests, Fig 4).

## Discussion

We documented significant patterns of spatio-temporal variation in the diversity and assemblage structure of understorey forest birds in the 10,000 ha Ducke Forest Reserve (DFR), central Amazonia over a three-year period, from 2012–2014. At the scale of this study, spatial differences in bird assemblages were related to environmental differences among plots and to topographic variables, such as slope and elevation, but were not related to purely spatial variables, suggesting that dispersal limitation is not operating strongly within the reserve for the majority of these birds at distances of ≤10 km. Despite clear differences in bird diversity and assemblage structure between eastern and western watersheds, we found no evidence to suggest an effect of the ridgeline *per se* on bird assemblages. Nonetheless, the central ridge may be acting as a boundary to limit the distribution of some species. The differences in bird assemblages between watersheds demonstrated how topographic variables may act alongside environmental variables to structure Amazonian forest bird assemblages. Our findings suggest that, at broader scales, what may often be detected as spatial structure (sensu [60]) might be due in part to biogeographic landscape features and associated natural boundaries in studies of multivariate assemblages.

### Temporal patterns

Alpha diversity tends to remain stable over time if environmental characteristics are also stable [64]. High spatial turnover of species in tropical forests is often explained by fine-scale variation in environmental conditions as well as stochastic processes yielding natural fluctuations in densities. If temporal turnover is caused by environmental changes, we would expect shifts in relative abundances of common species. To the contrary, we found higher temporal turnover of the more infrequently-captured species that may be rare or may occur in naturally low densities at the spatial scale and extent of our study (S4 Fig). This could be due to lower probabilities of detection for rare taxa (e.g [65]), larger home ranges or higher mobility of some of those species.

We consider that temporal changes in species composition were likely due to natural inter-annual stochastic processes rather than changes in environmental conditions. Longer-term studies, allowing rigorous estimation of detection probabilities, as well as studies at larger spatial scales are needed in order to develop more refined models of temporal changes in this system, particularly if indeed driven primarily by variations in occurrence and detection of rarer taxa.

### Spatial patterns

Our results indicate that spatial variation in bird diversity and assemblage structure in DFR was due to environmental heterogeneity among plots, represented by three environmental groups (Low, High and Slope areas). The three environmental groups defined here reflect topo-edaphic variation and differences in vegetation structure. Topographic heterogeneity in central Amazonia varies with soil texture, nutrient concentration and its underlying geology [38, 66]. Several studies have shown that topo-edaphic gradients are key drivers of plant and animal distributions in Amazon forests [12, 31, 32, 43, 67–71]. In addition, palm density is higher on sandy soils on valley bottoms, whereas tree density tends to be higher on clay soils along ridges [72]. As topo-edaphic variables determine plant structure and floristic composition, it is not surprising that they affect bird assemblages as well, as vegetation composition likely constitutes the most important feature of the forest for birds [31, 73, 74].

Areas at higher elevations were more diverse than other areas, and contained a distinct bird assemblage. Plots located on higher areas in DFR have higher densities of trees, so prey availability, and places to nest and hide, may be more abundant in these areas. The range of niches created by trees might support a greater diversity of species. Although we found that some mixed-species flocking birds were more abundant, on average, in High areas, in a neighboring reserve, Potts et al. [75] found that mixed-species flocks were more likely to move towards lower areas and used higher areas less frequently. While possible variation in movement related to topography was not the main focus of this study, these contradictory results suggest that understorey mixed-species flocks may behave differently in different regions of Amazonia.

Low areas also supported a distinct bird assemblage, characterized by species that are found more frequently in riparian habitats. Previous studies have shown that areas close to streams have distinct bird assemblages, with more species being habitat-restricted than those found in slope or ridge areas [32, 43]. Areas closer to streams are likely to be preferred by insectivorous birds because moister environments tend to hold higher abundances of arthropods [76, 77]. Other species, such as kingfishers (*Chloroceryle aenea*, *Chloroceryle inda*), also depend on streams for fish and were found only in Low areas.

Slope areas connect Low and High areas, thus are less distinct and possess environmental characteristics of both types of areas. Nonetheless, some frugivorous birds were more prevalent in Slope areas. As tree mortality is higher on steeper slopes in the DFR [39, 72], it is expected that Slope areas have more tree-fall gaps, which in turn, have higher incidence of fruiting plants to sustain frugivores [78–80].

Although comparisons among environmental groups may be influenced by the method (e.g., biases in mist-netting might vary with changes in the density of vegetation across the study area), we strongly believe that such forest features are more likely to affect the distribution of the birds rather than create detectable differences in capture rates between plots.

### Eastern and western watersheds carry distinct bird assemblages

The eastern and western watersheds supported significantly different bird assemblages. Although the two watersheds shared most of the species, three species were observed only in the western watershed and three others were observed only in the eastern watershed. While the detection of particular species in one or the other watershed might vary, due to variation in sampling biases associated with mist-netting in different environments, we nevertheless found that the eastern watershed had higher densities of individuals, on average and more diverse bird assemblages than the western watershed. Similar results have been found for shrubs [68], herbs [67], fish [81], frogs [82] and palms [69] in the same region. It is difficult to identify factors that may account for these differences. At the plot level, there is no clear overall change in soil or other environmental features between the two watersheds, but the two watersheds have different proportions of high and low-elevation areas, and birds may be responding to general landscape features rather than to individual plot characteristics.

Higher average numbers of species captured per plot, which presumably reflects species richness, in the eastern watershed may also be due to greater connectivity with a diverse forest bird community beyond the eastern and northern boundaries of the reserve. In contrast, on the western side there is less connectivity, due to deforestation and urbanization, resulting in an impoverished bird community beyond the eastern and southern boundaries of the reserve. Although most species occurred throughout the DFR, the central ridge may be avoided by birds—and hence infrequently crossed. That is, species restricted to one watershed or the other may be displaying a boundary response [83], i.e., a tendency to avoid leaving favorable habitat when the ridge is encountered, even if the ridge is not a physical barrier *per se*. For instance, species that are restricted to riparian habitats may find more corridors for movement on one side of the ridge, and thus exhibit fewer cross-ridge movements. This may be the case for species associated with riparian habitats, such as *S*. *leucostigma*, *C*. *inda* and *C*. *aenea*, captured only in the western watershed, which has a higher proportion of streams. Non-random movements driven by species’ perception of the landscape can shape bird assemblages [84–86]. Also, ecomorphological characteristics, such as reproductive potential, sociability, diet, body size and ecological specialization, are known to affect dispersal of birds [87–91], and could also contribute to bird compositional differences between watersheds.

### Limitations of the study

As each species in megadiverse tropical forests has its own requirements and behavior, identifying processes underlying observed spatio-temporal patterns is a challenging task for community ecologists. Complex combinations of factors can act simultaneously and interact to affect individual species. All environmental variables together explained less than 20% of the variation in species composition. Thus, there are likely to be many biological and environmental features important to birds that have not been measured or accounted for here. Potentially important unmeasured variables include canopy openness [30, 92], floristic composition [73, 74, 93] and food availability [76], all of which are known to affect bird distributions. Tree-fall gaps are also known to affect forest-bird richness and composition [78, 94]. Interestingly, few other studies attempting to model natural variation in tropical plant and animal assemblages could explain more than ~25% of the observed total variation [8, 12, 31, 95]–but see [9, 29, 96] for counterexamples. Thus, a large proportion of unexplained variation is typical of tropical-forest studies and is often attributable to low recorded densities for most species, combined with high stochastic turnover in time and space. Low dominance and a high proportion of rare species were also clearly apparent in the present study. Additional data—a greater number of sites as well as a number of landscape-level replicates, with more detailed measures at smaller scales of other potential environmental and biological predictors—are needed to help develop a more sophisticated understanding of the complex mechanisms that drive Amazonian forest-bird diversity. Also, analyses using multiple methods for data-collection (e.g., combining mist-nets with point counts) may help removing potential biases or artifacts associated with the use of a particular sampling method [28, 32].

### Implications for conservation

Overall, the avifauna we found was not substantially different from that encountered in past surveys in the DFR and in neighboring areas [32, 43, 44]. Although most species we captured are expected to be found in a healthy old-growth forest, the presence of non-forest species such as *Thryothorus coraya* and *Troglodytes aedon* suggests that there could be impacts of urbanization within DRF limits. The western and southern borders of DFR are surrounded by urban sprawl from the city of Manaus. Outside DFR’s limits, the environment is unsuitable; areas through which forest birds may safely disperse no longer exist. Over time, degradation of the western watershed is expected, due to the edge effects of urbanization, hunting, dogs and cats, and vegetation change [97–99]. Traffic noise is audible from the middle of the reserve (JM, *personal observation*); noise pollution may affect bird song, behavior and distribution [100, 101], a topic which should be studied at DFR. Long-term monitoring of birds and other taxa is warranted to follow the state of the assemblages in years to come; human impacts may then be quantified by comparing sites well within the reserve to those near borders. The eastern limits of DFR still have some connection to continuous forest, allowing birds to disperse to the east. It is likely that this connection will be a key factor in the maintenance of biodiversity in the future for the DFR. Connectivity may be maintained through development and protection of forest corridors allowing dispersal between the DFR and forest areas to the north-east of Manaus.

Our study indicates that complex topography and landscape features can act together with environmental variables to drive changes in the diversity and composition of tropical bird assemblages at local scales, but the weak explanatory power of measured variables highlights that we still know very little about what makes tropical forest suitable for different species. Nonetheless, assemblage differentiation between watersheds demonstrates that not only plot characteristics, but also landscape features require quantification. Useful models for long-term biodiversity management will need to incorporate local topography and its potential effects on the movement and connectivity of species into the spatial design of reserves for conservation purposes, as the sensitivity of species to even small-scale features may have important effects. Moreover, explicitly delimiting both protected areas and corridors among them will allow for individual movements, minimizing the potential for fragmentation and isolation of tropical bird assemblages.

## Supporting information

S1 Table Summary of environmental predictors (mean ± standard deviation, minimum and maximum) for eastern and western watersheds at Ducke Forest Reserve. Data obtained from ppbio.inpa.gov.br/repositorio/dados on 03/26/2015).
(PDF)

S2 Table List of bird species captured in 72 plots of the Ducke Forest Reserve in 2012, 2013 and 2014. Taxonomy and systematic order follow the IOC World Bird List (v 7.1).
(PDF)

S3 Table PERMANOVA pairwise results. Effects of ‘Environmental group’ and ‘Year’ on bird assemblage structure.
(PDF)

S4 Table Results of DISTLM sequential tests *within* sets of predictors. Cumulative proportion of variation (*R*^2^) in bird assemblage structure that is explained by fitting variables *within* sets sequentially using forward selection, and conditional tests using 9999 permutations of residuals under a reduced model. Values in bold indicate *P* < 0.10.
(PDF)

S1 Fig Principal Component Analysis (PCA) on environmental predictors. The colors indicate the three environmental groups identified by K-means partitioning. The closer the points are the more similar plots are in terms of their environmental variables.
(PDF)

S2 Fig Distribution of bird species in relation to watersheds in the Ducke Forest Reserve. Bars represent the presence of each species in a given plot. Orange bars are plots located in the eastern watershed (*n* = 34) while green bars represent plots located in the western watershed (*n* = 38).
(PDF)

S3 Fig Correlation of bird species with environmental groups. CAP ordination of bird data (based on *m* = 13 PCO axes) maximizing differences among the three *a priori* environmental groups, showing vector overlay of Pearson correlations of individual bird species with CAP axes (restricted to those having lengths > 0.25).
(PDF)

S4 Fig Distribution of bird species over the years in the Ducke Forest Reserve. Bars represent the presence of each species in a given plot. Pale blue dots represent 2012; green dots represent 2013 and dark blue dots represent 2014.
(PDF)

S1 File Results of analyses using “Common forest understorey species” dataset. Analyses were based on 64 bird species.
(PDF)
