# Supplementary material for: Environmental characteristics drive variation in Amazonian understorey bird assemblages
Source: PLoS One. 2017 Feb 22;12(2):e0171540. doi: 10.1371/journal.pone.0171540 (PMC5321421; doi:10.1371/journal.pone.0171540)
Supplement: S1 Table — Data obtained from ppbio.inpa.gov.br/repositorio/dados on 03/26/2015). (PDF) [file pone.0171540.s001.pdf]

S1 Table. Summary of environmental predictors (mean ± standard deviation, minimum and maximum) for eastern and western watersheds at Ducke Forest Reserve. Data obtained from ppbio.inpa.gov.br/repositorio/dados on 03/26/2015).

	Western			Eastern		
**Variable**	**Mean (±SD)**	**Min**	**Max**	**Mean (±SD)**	**Min**	**Max**
[1]**Elevation (m)**	75.89 (19.54)	46.79	109.77	76.25 (20.40)	39.39	102.23
[2]**Slope (degree)**	10.01 (6.80)	0.67	26.33	9.95 (8.01)	0.67	27
[3]**Distance to stream**	215.81 (161.07)	18.22	579.18	255.22 (161.87)	9.87	602.92
[4]**Clay content (%)**	41.22 (33.37)	1.62	86.30	54.39 (31.34)	7.16	87.74
[4]**Silt content (%)**	3.20 (1.75)	0.29	9.28	3.55 (2.20)	0.72	14.15
[5]**Palm density (ind ha^{-1})**	183.76 (127.63)	0	475	85.41 (81.38)	2	308
[5]**Tree density (ind ha^{-1})**	4423.63 (979.80)	2670	6430	5401.32 (1040.29)	4301	8707

[1]Luizão F, Luizão R & Magnusson WE. Access code: fecosta.25.2; [2]Lima AP & Castilho CV. Access code: fecosta.26.8; [3]Schietti J. Access code: pezzini.83.6; [4]Luizão F. Access code: fecosta.31.2; [5]Castilho CV & Araújo RNO. Access code: melo.33.18
