# Supplementary material for: Environmental characteristics drive variation in Amazonian understorey bird assemblages
Source: PLoS One. 2017 Feb 22;12(2):e0171540. doi: 10.1371/journal.pone.0171540 (PMC5321421; doi:10.1371/journal.pone.0171540)
Supplement: S2 Table — Taxonomy and systematic order follow the IOC World Bird List (v 7.1). (PDF) [file pone.0171540.s002.pdf]

S2 Table. List of bird species captured in 72 plots of the Ducke Forest Reserve in 2012, 2013 and 2014. Taxonomy and systematic order follow the IOC World Bird List v.7.1 [1].

Species by family	Primary stratum[1]	Primary habitat[1]	Western		Eastern	
			n Plot	*n* Individual	*n* Plot	*n* Individual
COLUMBIDAE						
Geotrygon montana (Linnaeus, 1758)	understorey	old-growth forest	10	14	7	8
STRIGIDAE						
Strix virgata (Cassin, 1849)	canopy	secondary forest	0	0	1	1
Glaucidium hardyi Vielliard, 1989	canopy	old-growth forest	0	0	1	1
TROCHILIDAE						
Phaethornis superciliosus (Linnaeus, 1766)	understorey	old-growth forest	21	49	18	58
Phaethornis bourcieri (Lesson, R, 1832)	understorey	old-growth forest	16	25	21	34
Campylopterus largipennis (Boddaert, 1783)	understorey	old-growth forest	5	6	2	2
Florisuga mellivora (Linnaeus, 1758)	canopy	old-growth forest	1	1	1	1

S3 Table. Continued

Thalurania furcata (Gmelin, JF, 1788)	understorey	old-growth forest	24	36	15	22
Amazilia versicolor (Vieillot, 1818)	canopy	secondary forest	0	0	1	1
TROGONIDAE						
Trogon rufus Gmelin, JF, 1788	understorey	old-growth forest	1	1	0	0
ALCEDINIDAE						
Chloroceryle aenea (Pallas, 1764)	understorey	old-growth forest	2	2	0	0
Chloroceryle inda (Linnaeus, 1766)	understorey	old-growth forest	2	2	0	0
MOMOTIDAE						
Momotus momota (Linnaeus, 1766)	midstorey	old-growth forest	6	8	12	16
GALBULIDAE						
Galbula albirostris Latham, 1790	understorey	old-growth forest	7	10	5	10
BUCCONIDAE						
Bucco tamatia Gmelin, JF, 1788	canopy	old-growth forest	1	1	1	1
Bucco capensis Linnaeus, 1766	midstorey	old-growth forest	2	3	1	1
Malacoptila fusca (Gmelin, JF, 1788)	understorey	old-growth forest	0	0	4	5

S3 Table. Continued

Nonnula rubecula (von Spix, 1824)	midstorey	old-growth forest	0	0	1	1
Monasa atra (Boddaert, 1783)	canopy	old-growth forest	2	2	0	0
RAMPHASTIDAE						
Pteroglossus viridis (Linnaeus, 1766)	canopy	old-growth forest	0	0	1	1
PICIDAE						
Veniliornis cassini (Malherbe, 1862)	canopy	old-growth forest	0	0	1	1
Celeus undatus (Linnaeus, 1766)	canopy	secondary forest	1	1	0	0
Celeus elegans (Statius Müller, PL, 1776)	canopy	old-growth forest	1	1	0	0
FALCONIDAE						
Micrastur ruficollis (Vieillot, 1817)	midstorey	old-growth forest	0	0	2	2
Micrastur gilvicollis (Vieillot, 1817)	midstorey	old-growth forest	2	2	2	4
FURNARIIDAE						
Philydor ruficaudatum (d'Orbigny & Lafresnaye, 1838)	midstorey	old-growth forest	1	1	1	1
Philydor erythrocercum (Pelzeln, 1859)	midstorey	old-growth forest	2	2	6	11
Automolus ochrolaemus (Tschudi, 1844)	understorey	secondary forest	1	2	1	3

S3 Table. Continued

Automolus infuscatus (Sclater, PL, 1856)	understorey	old-growth forest	3	3	2	4
Sclerurus mexicanus Sclater, PL, 1857	understorey	old-growth forest	4	4	6	8
Sclerurus rufigularis Pelzeln, 1868	understorey	old-growth forest	9	12	9	12
Sclerurus caudacutus (Vieillot, 1816)	understorey	old-growth forest	0	0	1	1
Xenops minutus (Sparrman, 1788)	understorey	old-growth forest	2	2	3	6
Dendrocincla fuliginosa (Vieillot, 1818)	understorey	old-growth forest	20	33	19	31
Dendrocincla merula (Lichtenstein, MHK, 1820)	understorey	old-growth forest	25	49	15	27
Deconychura longicauda (Pelzeln, 1868)	understorey	old-growth forest	7	8	10	15
Deconychura stictolaema (Pelzeln, 1868)	understorey	old-growth forest	10	13	12	16
Sittasomus griseicapillus (Vieillot, 1818)	canopy	old-growth forest	1	1	1	1
Glyphorynchus spirurus (Vieillot, 1819)	midstorey	old-growth forest	34	114	34	165
Hylexetastes perrotii (Lafresnaye, 1844)	midstorey	old-growth forest	8	10	1	1
Dendrocolaptes certhia (Boddaert, 1783)	midstorey	old-growth forest	1	1	3	3
Dendrocolaptes picumnus Lichtenstein, MHK, 1820	midstorey	old-growth forest	0	0	1	1
Xiphorhynchus pardalotus (Vieillot, 1818)	understorey	old-growth forest	29	61	27	55

S3 Table. Continued

Campylorhamphus procurvoides (Lafresnaye, 1850)	midstorey	old-growth forest	1	1	4	7
THAMNOPHILIDAE						
Cymbilaimus lineatus (Leach, 1814)	midstorey	secondary forest	1	1	0	0
Frederickena viridis (Vieillot, 1816)	understorey	old-growth forest	9	15	9	12
Thamnophilus murinus Sclater, PL & Salvin, 1868	midstorey	old-growth forest	5	5	7	10
Thamnomanes ardesiacus (Sclater, PL & Salvin, 1868)	understorey	old-growth forest	8	22	13	37
Thamnomanes caesius (Temminck, 1820)	understorey	old-growth forest	12	20	13	39
Epinecrophylla gutturalis (Sclater, PL & Salvin, 1881)	understorey	old-growth forest	12	23	11	28
Myrmotherula longipennis Pelzeln, 1868	understorey	old-growth forest	2	5	4	7
Myrmotherula menetriesii (d'Orbigny, 1837)	understorey	old-growth forest	5	11	5	9
Hypocnemis cantator (Boddaert, 1783)	understorey	old-growth forest	14	37	19	42
Percnostola rufifrons (Gmelin, JF, 1789)	understorey	old-growth forest	18	38	24	47
Schistocichla leucostigma (Pelzeln, 1868)	understorey	old-growth forest	8	9	0	0
Myrmeciza ferruginea (Statius Müller, PL, 1776)	understorey	old-growth forest	7	12	7	9
*Myrmeciza atrothorax (*Boddaert, 1783)	understorey	secondary forest	1	1	0	0

S3 Table. Continued

Pithys albifrons (Linnaeus, 1766)	understorey	old-growth forest	28	104	27	87
Gymnopithys rufigula (Boddaert, 1783)	understorey	old-growth forest	23	55	18	64
Willisornis poecilinotus (Cabanis, 1847)	understorey	old-growth forest	5	9	8	12
FORMICARIIDAE						
Formicarius colma Boddaert, 1783	understorey	old-growth forest	10	14	18	30
CONOPOPHAGIDAE						
Conopophaga aurita (Gmelin, JF, 1789)	understorey	old-growth forest	0	0	1	1
TYRANNIDAE						
Mionectes oleagineus (Lichtenstein, MHK, 1823)	understorey	old-growth forest	6	7	10	10
Mionectes macconnelli (Chubb, C, 1919)	understorey	old-growth forest	30	70	26	54
Hemitriccus zosterops (Pelzeln, 1868)	midstorey	old-growth forest	1	1	0	0
Rhynchocyclus olivaceus (Temminck, 1820)	midstorey	old-growth forest	2	2	1	1
Tolmomyias assimilis (Pelzeln, 1868)	canopy	old-growth forest	0	0	3	3
Platyrinchus saturatus Salvin & Godman, 1882	understorey	old-growth forest	10	10	11	14
Platyrinchus platyrhynchos (Gmelin, JF, 1788)	midstorey	old-growth forest	1	1	1	1

S3 Table. Continued

Rhytipterna simplex (Lichtenstein, MHK, 1823)	canopy	old-growth forest	3	3	0	0
Attila spadiceus (Gmelin, JF, 1789)	canopy	old-growth forest	11	17	10	13
COTINGIDAE						
Phoenicircus carnifex (Linnaeus, 1758)	canopy	old-growth forest	1	1	0	0
Lipaugus vociferans (zu Wied-Neuwied, 1820)	canopy	old-growth forest	0	0	1	1
PIPRIDAE						
Corapipo gutturalis (Linnaeus, 1766)	understorey	old-growth forest	2	2	6	6
Lepidothrix serena (Linnaeus, 1766)	understorey	old-growth forest	10	20	9	14
Manacus manacus (Linnaeus, 1766)	understorey	secondary forest	1	1	0	0
Pseudopipra pipra (Linnaeus, 1758)	understorey	old-growth forest	28	77	31	104
Ceratopipra erythrocephala (Linnaeus, 1758)	midstorey	old-growth forest	2	2	3	4
TITYRIDAE						
Onychorhynchus coronatus (Statius Müller, PL, 1776)	understorey	old-growth forest	6	8	5	5
Myiobius barbatus (Gmelin, JF, 1789)	midstorey	old-growth forest	0	0	4	5
Schiffornis turdina (zu Wied-Neuwied, 1831)	understorey	old-growth forest	2	3	1	1

S3 Table. Continued

Laniocera hypopyrra (Vieillot, 1817)	midstorey	old-growth forest	0	0	1	1
Pachyramphus marginatus (Lichtenstein, MHK, 1823)	midstorey	old-growth forest	1	2	0	0
VIREONIDAE						
Hylophilus ochraceiceps Sclater, PL, 1860	understorey	old-growth forest	5	10	5	13
TROGLODYTIDAE						
Pheugopedius coraya (Gmelin, JF, 1789)	understorey	secondary forest	3	3	4	6
Troglodytes aedon Vieillot, 1809	understorey	secondary forest	1	1	0	0
Microcerculus bambla (Boddaert, 1783)	understorey	old-growth forest	2	2	3	3
Cyphorhinus arada (Hermann, 1783)	understorey	old-growth forest	3	3	2	7
POLIOPTILIDAE						
Microbates collaris (Pelzeln, 1868)	understorey	old-growth forest	1	1	2	2
TURDIDAE						
Turdus albicollis Vieillot, 1818	understorey	old-growth forest	18	31	18	39
ICTERIDAE						
Cacicus solitarius Vieillot, 1816	midstorey	secondary forest	0	0	1	1

S3 Table. Continued

Cacicus haemorrhous (Linnaeus, 1766)	canopy	old-growth forest	1	2	0	0
EMBEREZIDAE						
Arremon taciturnus (Hermann, 1783)	understorey	old-growth forest	1	1	2	2
THRAUPIDAE						
Tachyphonus cristatus (Linnaeus, 1766)	canopy	old-growth forest	1	1	0	0
Tachyphonus surinamus (Linnaeus, 1766)	understorey	old-growth forest	14	27	10	12
Lanio fulvus (Boddaert, 1783)	midstorey	old-growth forest	3	3	2	2
Saltator grossus (Linnaeus, 1766)	canopy	old-growth forest	1	1	0	0
CARDINALIDAE						
Cyanocompsa cyanoides (Lafresnaye, 1847)	understorey	secondary forest	5	7	4	5

[1]Preferred stratum and habitat following [2].

1. Gill F, Donsker D. 2017. IOC World Bird List (v 7.1). doi: 10.14344/IOC.ML.7.1.

2. Cohn-Haft M, Whittaker A, Stouffer PC. A new look at the "species-poor" central Amazon: the avifauna north of Manaus, Brazil. Ornithological Monographs. 1997;48:205-35. doi: 10.2307/40157535.
