# Supplementary material for: Environmental characteristics drive variation in Amazonian understorey bird assemblages
Source: PLoS One. 2017 Feb 22;12(2):e0171540. doi: 10.1371/journal.pone.0171540 (PMC5321421; doi:10.1371/journal.pone.0171540)
Supplement: S3 Table — Effects of ‘Environmental group’ and ‘Year’ on bird assemblage structure. (PDF) [file pone.0171540.s003.pdf]

S3 Table. PERMANOVA pairwise results. Effects of 'Environmental group' and 'Year' on bird assemblage structure. PERMANOVA pairwise tests with *p*-values obtained using 9999 permutations under a reduced model. Values in bold indicate a significant effect.

Environmental group			Year		
**Comparison**	***t***	***P***	**Comparison**	***t***	***P***
Low *vs* High	1.609	**0.0007**	2012 *vs* 2013	1.067	0.3320
Low *vs* Slope	1.493	**0.0045**	2012 *vs* 2014	1.385	**0.0190**
Slope *vs* High	2.004	**0.0001**	2013 *vs* 2014	1.220	0.1035
