# Supplementary material for: Environmental characteristics drive variation in Amazonian understorey bird assemblages
Source: PLoS One. 2017 Feb 22;12(2):e0171540. doi: 10.1371/journal.pone.0171540 (PMC5321421; doi:10.1371/journal.pone.0171540)
Supplement: S4 Table — Cumulative proportion of variation (R2) in bird assemblage structure that is explained by fitting variables within sets sequentially using forward selection, and conditional tests using 9999 permutations of residuals under a reduced model. Values in bold indicate P < 0.10. (PDF) [file pone.0171540.s004.pdf]

S4 Table. Results of DISTLM sequential tests *within* sets of predictors. Cumulative proportion of variation (R^2) in bird assemblage structure that is explained by fitting variables *within* sets sequentially using forward selection, and conditional tests using 9999 permutations of residuals under a reduced model. Values in bold indicate $P < 0.10$.

	**pseudo-*F***	***P***	**cumulative R^2**
**ENVIRONMENTAL VARIABLES**			
dist. stream	2.954	**0.0001**	0.0405
clay	2.299	**0.0015**	0.0714
tree	1.568	**0.0605**	0.0924
silt	1.206	0.2415	0.1084
palm	1.089	0.3660	0.1229
**TOPOGRAPHIC VARIABLES**			
elevation	2.728	**0.0004**	0.0375
slope	2.351	**0.0016**	0.0692
watershed	1.498	**0.0788**	0.0893
**SPATIAL VARIABLES**			
y^2	1.607	**0.0474**	0.0224
x	1.196	0.2598	0.0391
x^3	0.773	0.7366	0.0499
x^2	1.077	0.3782	0.0649
y^3	0.646	0.8649	0.0740
y	0.787	0.7269	0.0851
yx^2	0.469	0.9654	0.0917
yx	0.849	0.6491	0.1038
y^2x	0.790	0.7225	0.1151

x and *y* refer to longitude, latitude and their polynomials up to 3rd order, respectively.
