# Supplementary material for: Environmental characteristics drive variation in Amazonian understorey bird assemblages
Source: PLoS One. 2017 Feb 22;12(2):e0171540. doi: 10.1371/journal.pone.0171540 (PMC5321421; doi:10.1371/journal.pone.0171540)
Supplement: S1 Fig — The colors indicate the three environmental groups identified by K-means partitioning. The closer the points are the more similar plots are in terms of their environmental variables. (PDF) [file pone.0171540.s005.pdf]

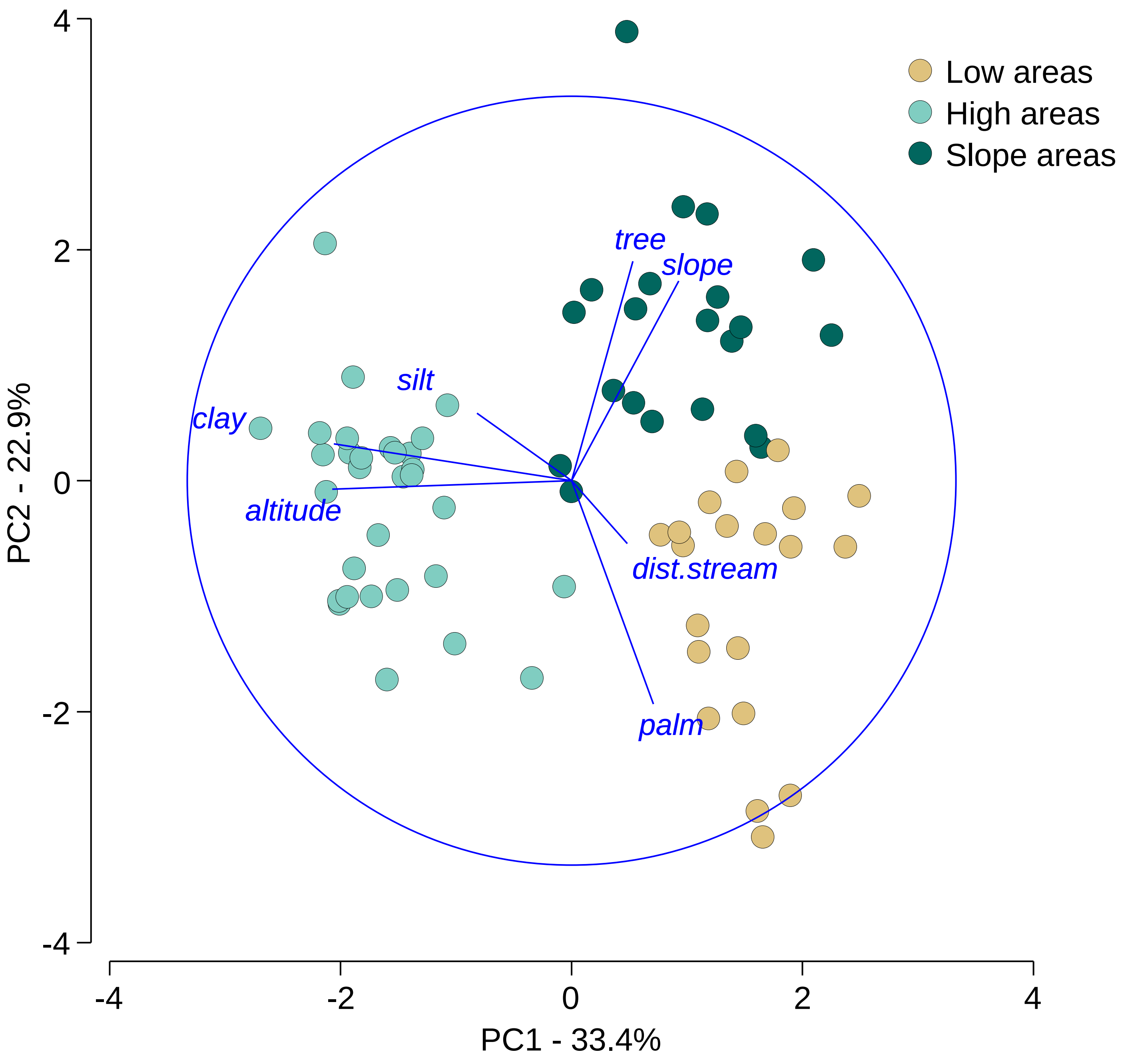


**S1 Fig. Principal Component Analysis (PCA) on environmental predictors.**

The colors indicate the three environmental groups identified by K-means partitioning. The closer the points are the more similar plots are in terms of their environmental variables.
