# Supplementary material for: Environmental characteristics drive variation in Amazonian understorey bird assemblages
Source: PLoS One. 2017 Feb 22;12(2):e0171540. doi: 10.1371/journal.pone.0171540 (PMC5321421; doi:10.1371/journal.pone.0171540)
Supplement: S2 Fig — Bars represent the presence of each species in a given plot. Orange bars are plots located in the eastern watershed (n = 34) while green bars represent plots located in the western watershed (n = 38). (PDF) [file pone.0171540.s006.pdf]

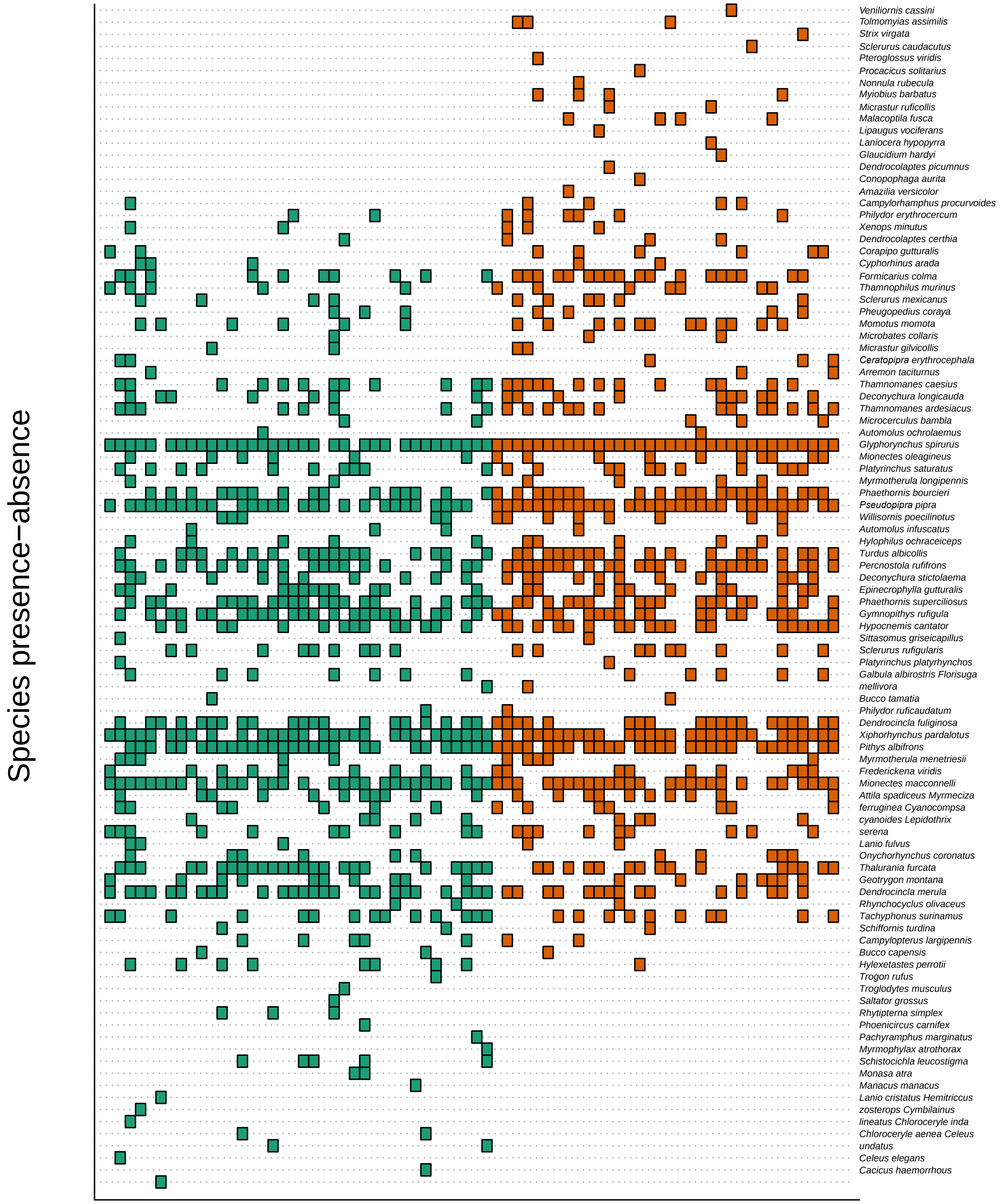

S2 Fig. Distribution of bird species in relation to watersheds in the Ducke Forest Reserve. Bars represent the presence of each species in a given plot. Orange bars are plots located in the eastern watershed (*n* = 34) while green bars represent plots located in the western watershed (*n* = 38).
