# Supplementary material for: Environmental characteristics drive variation in Amazonian understorey bird assemblages
Source: PLoS One. 2017 Feb 22;12(2):e0171540. doi: 10.1371/journal.pone.0171540 (PMC5321421; doi:10.1371/journal.pone.0171540)
Supplement: S3 Fig — CAP ordination of bird data (based on m = 13 PCO axes) maximizing differences among the three a priori environmental groups, showing vector overlay of Pearson correlations of individual bird species with CAP axes (restricted to those having lengths > 0.25). (PDF) [file pone.0171540.s007.pdf]

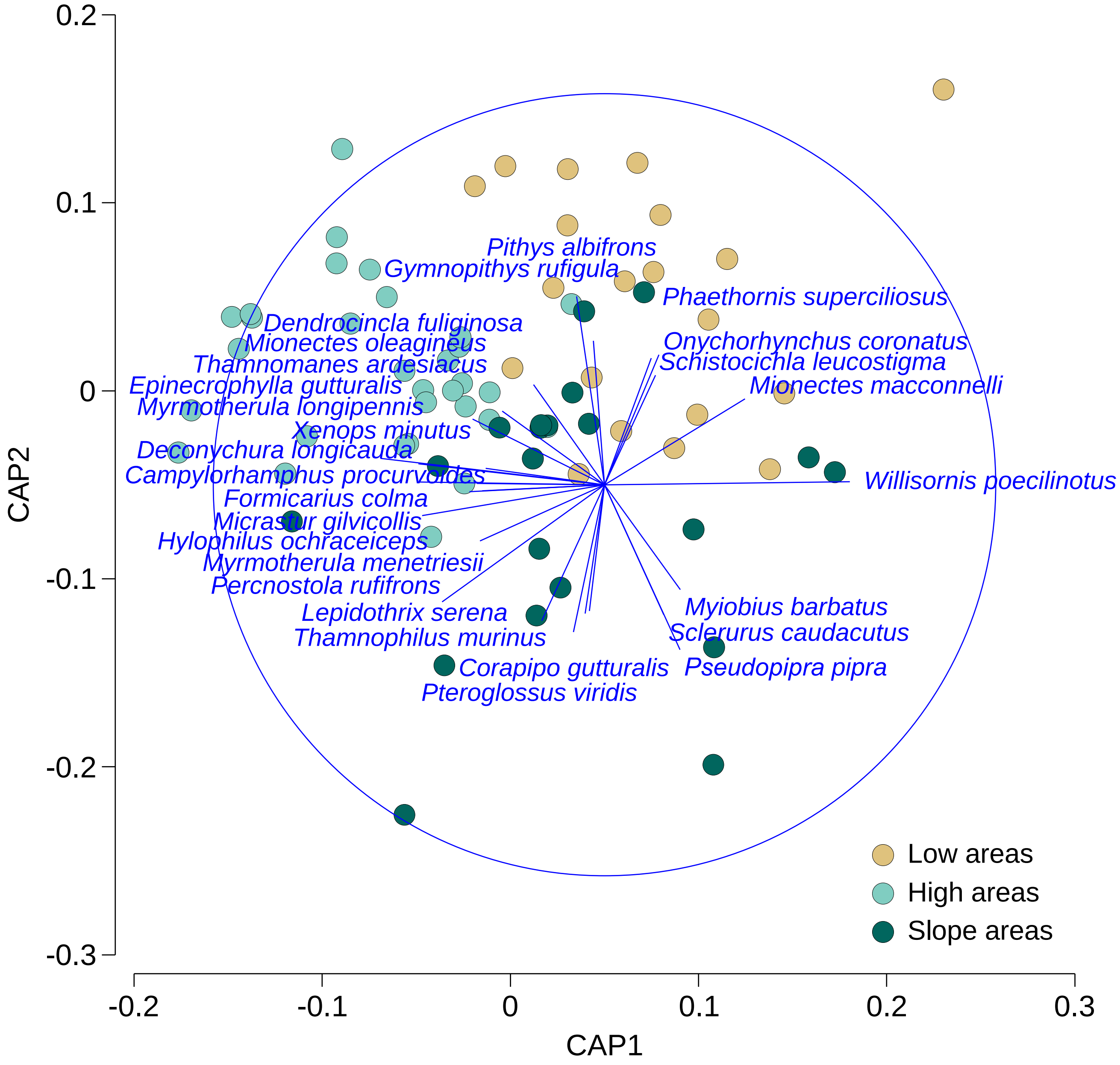

S3 Fig. Correlation of bird species with environmental groups. CAP ordination of bird data (based on m = 13 PCO axes) maximizing differences among the three *a priori* environmental groups, showing vector overlay of Pearson correlations of individual bird species with CAP axes (restricted to those having lengths > 0.30).
