# Supplementary material for: Environmental characteristics drive variation in Amazonian understorey bird assemblages
Source: PLoS One. 2017 Feb 22;12(2):e0171540. doi: 10.1371/journal.pone.0171540 (PMC5321421; doi:10.1371/journal.pone.0171540)
Supplement: S4 Fig — Bars represent the presence of each species in a given plot. Pale blue dots represent 2012; green dots represent 2013 and dark blue dots represent 2014. (PDF) [file pone.0171540.s008.pdf]

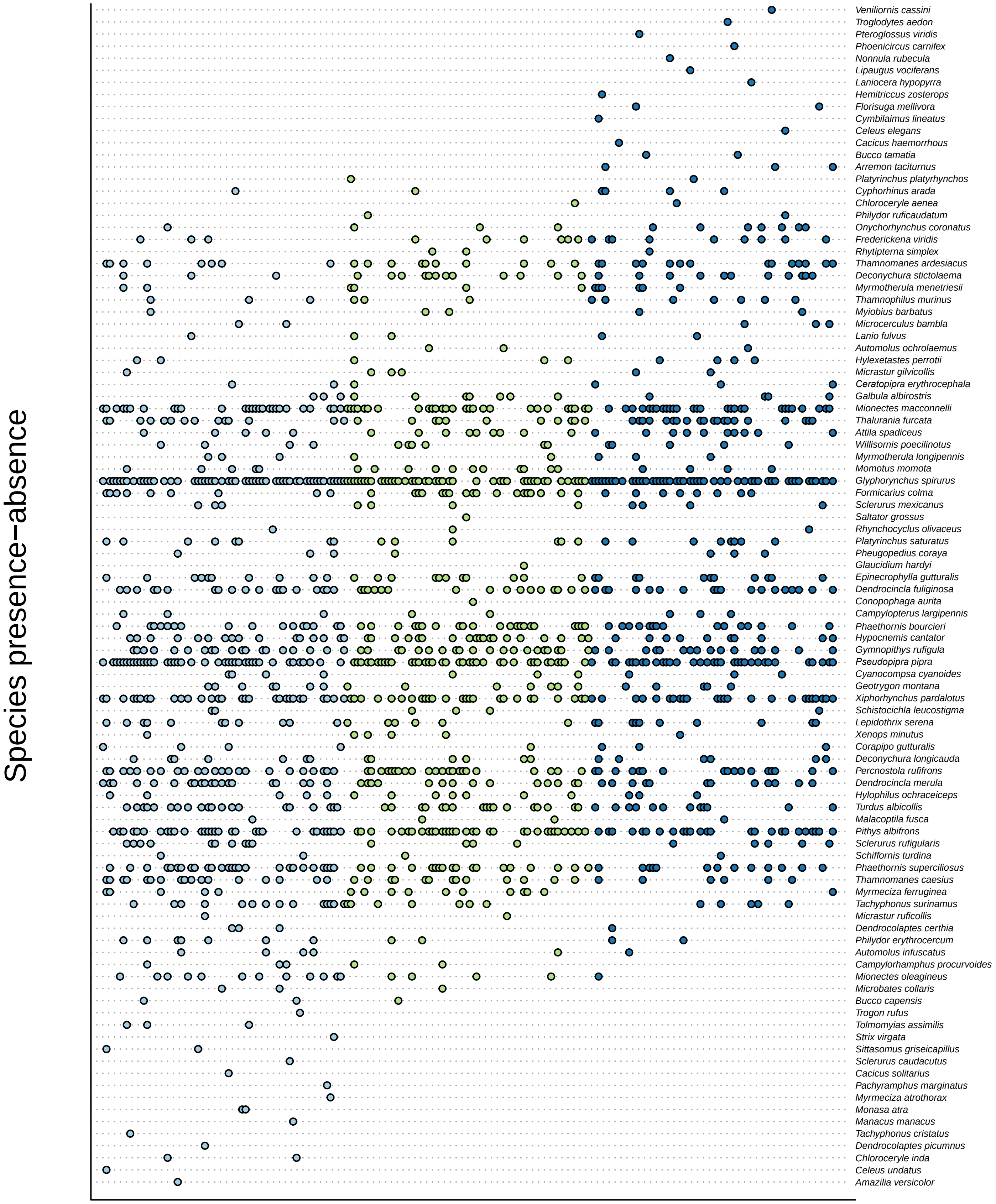

S4 Fig. Distribution of bird species over the years in the Ducke Forest Reserve. Dots represent the presence of each species in a given plot. Pale blue dots represent 2012; green dots represent 2013 and dark blue dots represent 2014.
