# Supplementary material for: Environmental characteristics drive variation in Amazonian understorey bird assemblages
Source: PLoS One. 2017 Feb 22;12(2):e0171540. doi: 10.1371/journal.pone.0171540 (PMC5321421; doi:10.1371/journal.pone.0171540)
Supplement: S1 File — Analyses were based on 64 bird species. (PDF) [file pone.0171540.s009.pdf]

S1 File. Results of analyses using “Common forest understorey species” dataset. Analyses were based on 64 bird species.

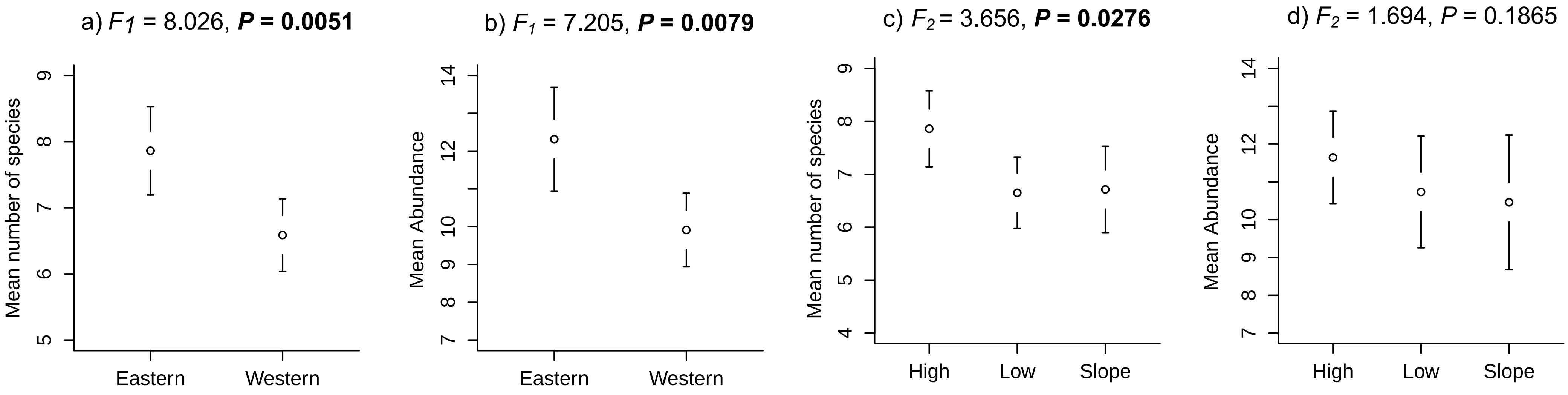

S1.1. Mean number of species and abundance of birds vs. environmental groups and watersheds. Mean number of species captured per plot or mean abundance in different watersheds (a and b, respectively) and in different environmental groups (c and d, respectively). Bars indicate 95% confidence intervals

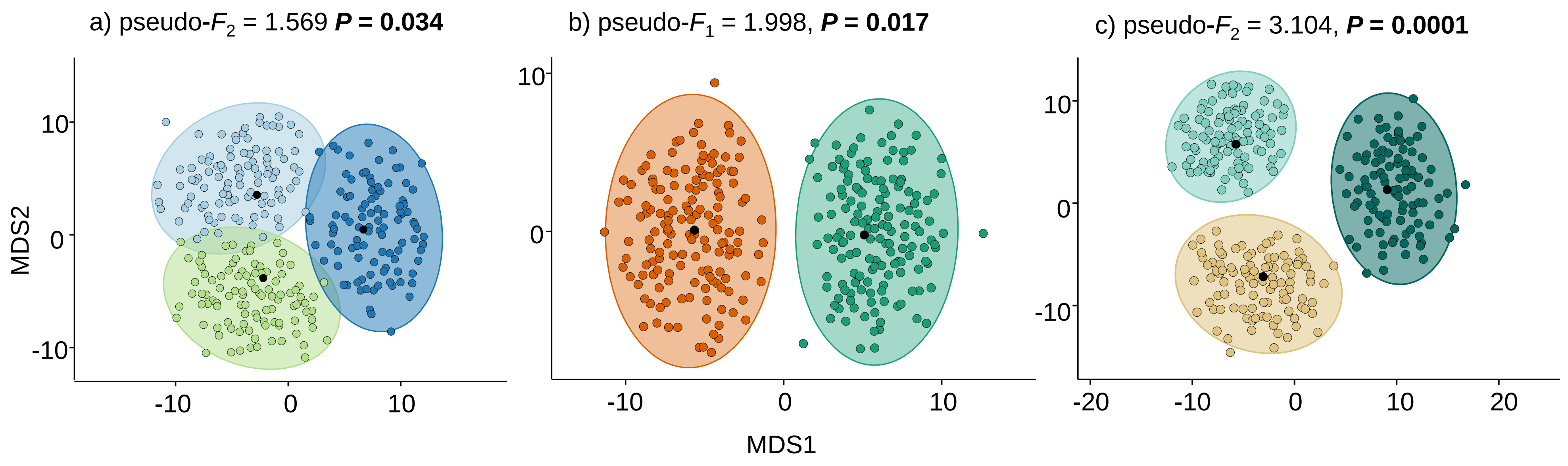

S1.2. **Ordinations of bootstrap averages of bird assemblages.** Two-dimensional metric multi-dimensional scaling ordinations of 100 bootstrap sample averages for each group for each of the main factors: Years (a), Watersheds (b); and Environmental groups (c), showing the overall mean (black dots) and the empirical approximate 95% confidence region, based on zero-adjusted Bray-Curtis dissimilarities of square-root transformed abundances of 64 bird species.

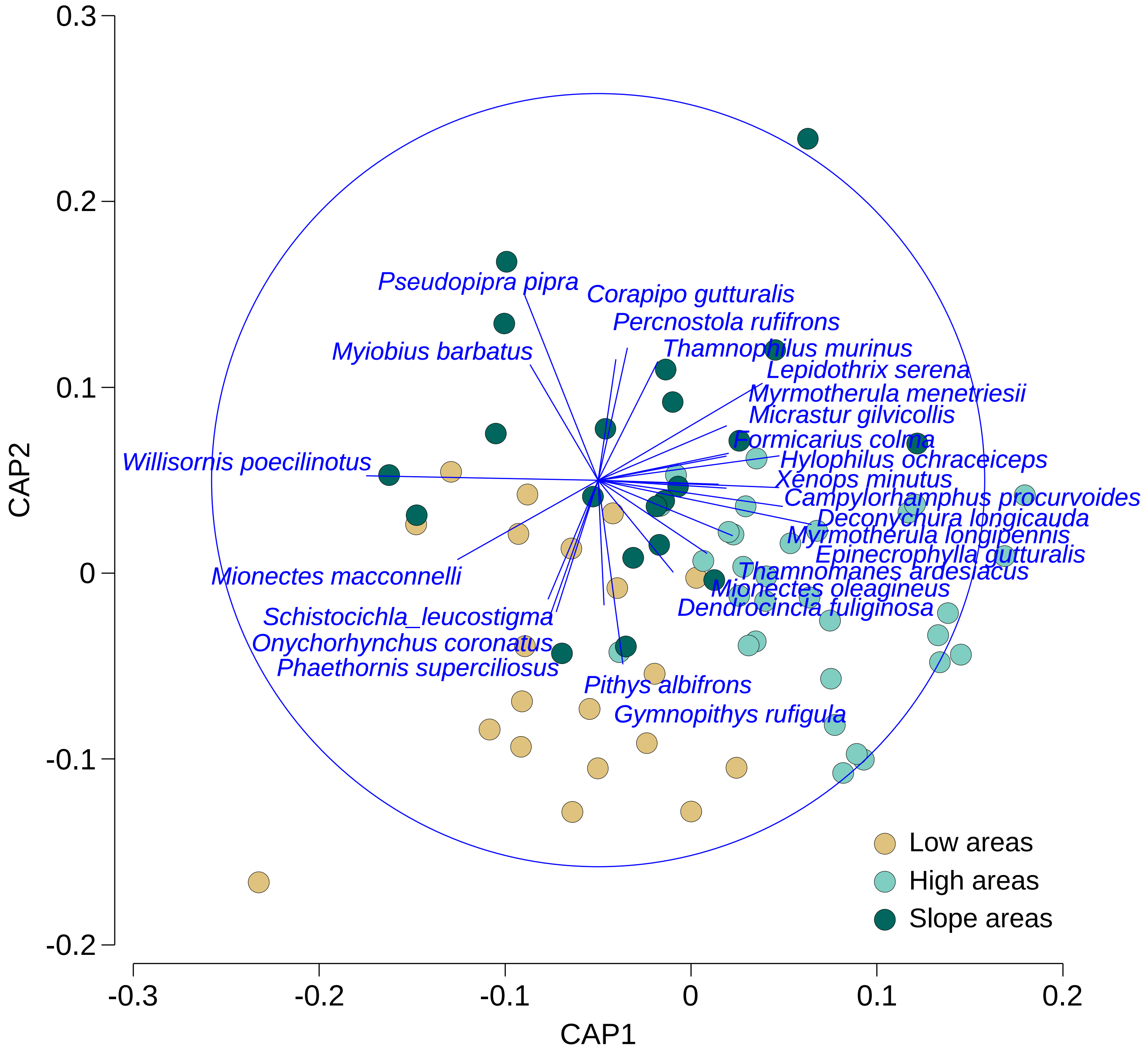

S1.3. Correlation of bird species with environmental groups. CAP ordination of bird data (based on *m* = 13 PCO axes) maximizing differences among the three a priori environmental groups, showing vector overlay of Pearson correlations of individual bird species with CAP axes (restricted to those having lengths > 0.30).

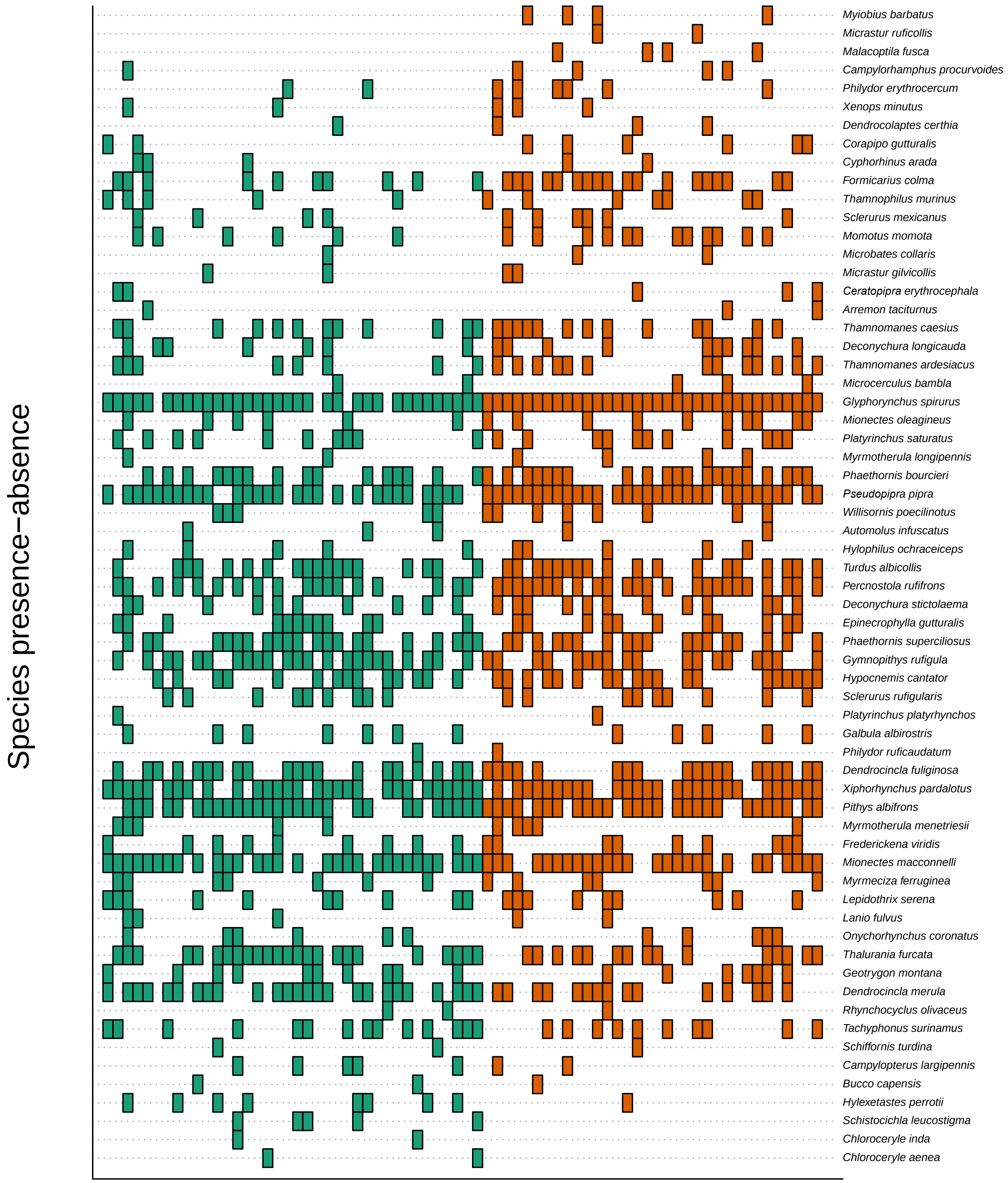

S1.4. Distribution of bird species in relation to watersheds in the Ducke Forest Reserve. Bars represent the presence of each species in a given plot. Orange bars are plots located in the eastern watershed (*n* = 34) while green bars represent plots located in the western watershed (*n* = 38).

S1.5. Results of DISTLM sequential tests *within* sets of predictors. Cumulative proportion of variation (R^2) in bird assemblage structure that is explained by fitting variables *within* sets sequentially using forward selection, and conditional tests using 9999 permutations of residuals under a reduced model. Values in bold indicate P < 0.10.

	**pseudo-*F***	***P***	**cumulative R^2**
**ENVIRONMENTAL VARIABLES**			
dist. stream	3.208	**0.0002**	0.0438
clay	2.389	**0.0008**	0.0758
tree	1.6805	**0.0406**	0.0981
silt	1.1619	0.2972	0.1135
palm	1.1371	0.3227	0.1285
**TOPOGRAPHIC VARIABLES**			
elevation	2.854	**0.0002**	0.0391
slope	2.416	**0.0020**	0.0717
watershed	1.600	**0.0587**	0.0930
**SPATIAL VARIABLES**			
y	1.646	**0.0519**	0.0229
x	1.284	0.1988	0.0408
x^2	0.782	0.7158	0.0517
x^3	1.146	0.3091	0.0676
y^3	0.628	0.8590	0.0764
y^2	0.688	0.8040	0.0861
yx^2	0.430	0.9677	0.0922
yx	0.816	0.6774	0.1038
y^2x	0.724	0.7760	0.1142

x and *y* refer to longitude, latitude and their polynomials up to 3rd order, respectively.

S1.6. Results of DISTLM analyses *among* sets of predictors. Proportion of variation (R^2) in bird assemblage structure that is explained by each set of variables when taken alone (marginal tests) and the cumulative proportion explained by fitting variables sequentially using forward selection. Values in bold indicate significant effect.

	MARGINAL TESTS				SEQUENTIAL TESTS		
**set**	**pseudo-*F***	***P***	**R^2**	**AICc**	**pseudo-*F***	***P***	**cumulative R^2**
**environmental**	2.4660	**0.0001**	0.0981	532.61	2.466	**0.0001**	0.0981
**topographic**	2.3247	**0.0001**	0.0930	533.01	1.6537	**0.0034**	0.1621
**spatial**	1.2401	0.1247	0.0519	536.21	1.1904	0.1804	0.2077

S1.7. Results of DISTLM analyses on all predictors. Proportion of variation (R^2) in bird assemblage structure (based on adjusted Bray-Curtis dissimilarities of square-root transformed abundances) explained by each predictor variable when taken alone (marginal tests) and the cumulative proportion explained by fitting variables sequentially using forward selection. Values in bold indicate $P < 0.1$.

	**MARGINAL TESTS**			**SEQUENTIAL TESTS**		
**variable**	**pseudo-*F***	***P***	**R^2**	**pseudo-*F***	***P***	**cumulative R^2**
**dist. stream**	3.208	**0.0001**	0.0438	3.208	**0.0001**	0.044
**elevation**	2.854	**0.0004**	0.0392	2.556	**0.0003**	0.078
**slope**	2.713	**0.0009**	0.0373	2.225	**0.0028**	0.107
**tree**	1.692	**0.0396**	0.0235	1.6801	**0.0398**	0.129
x	1.252	0.2195	0.0176	1.6045	**0.0590**	0.149
**watershed**	1.533	**0.0803**	0.0214	1.4135	0.1196	0.168
**silt**	1.213	0.2437	0.0170	1.3842	0.1429	0.185
**clay**	2.737	**0.0003**	0.0376	1.1355	0.3236	0.199
x^3	1.103	0.3578	0.0155	1.030	0.4286	0.213
**palm**	1.707	**0.0366**	0.0238	0.999	0.4757	0.226
y^3	1.625	**0.0511**	0.0227	0.979	0.4840	0.238
x^2	1.761	0.2870	0.0165	0.816	0.6748	0.248
y	1.646	**0.0479**	0.0229	0.823	0.6625	0.259
y^2	1.639	**0.0486**	0.0229	0.773	0.7230	0.269
yx^2	1.202	0.2625	0.0169	0.528	0.922	0.276
y^2x	1.2864	0.1978	0.0180	0.708	0.7843	0.285
yx	1.4044	0.1238	0.0197	1.108	0.3395	0.299

x and *y* refer to longitude, latitude and their polynomials up to 3rd order, respectively.
